# Does the eHealth Literacy Scale (eHEALS) Measure What it Intends to Measure? Validation of a Dutch Version of the eHEALS in Two Adult Populations

**DOI:** 10.2196/jmir.1840

**Published:** 2011-11-09

**Authors:** Rosalie van der Vaart, Alexander JAM van Deursen, Constance HC Drossaert, Erik Taal, Jan AMG van Dijk, Mart AFJ van de Laar

**Affiliations:** ^1^Department of Psychology, Health and TechnologyUniversity of TwenteEnschedeNetherlands; ^2^Department of Media, Communication and OrganizationUniversity of TwenteEnschedeNetherlands; ^3^Arthritis Centre TwenteEnschedeNetherlands

**Keywords:** e-health, literacy, internet, online, skills, health care, information

## Abstract

**Background:**

The Internet increases the availability of health information, which consequently expands the amount of skills that health care consumers must have to obtain and evaluate health information. Norman and Skinner in 2006 developed an 8-item self-report eHealth literacy scale to measure these skills: the eHealth Literacy Scale (eHEALS). This instrument has been available only in English and there are no data on its validity.

**Objectives:**

The objective of our study was to assess the internal consistency and the construct and predictive validity of a Dutch translation of the eHEALS in two populations.

**Methods:**

We examined the translated scale in a sample of patients with rheumatic diseases (n = 189; study 1) and in a stratified sample of the Dutch population (n = 88; study 2). We determined Cronbach alpha coefficients and analyzed the principal components. Convergent validity was determined by studying correlations with age, education, and current (health-related) Internet use. Furthermore, in study 2 we assessed the predictive validity of the instrument by comparing scores on the eHEALS with an actual performance test.

**Results:**

The internal consistency of the scale was sufficient: alpha = .93 in study 1 and alpha = .92 in study 2. In both studies the 8 items loaded on 1 single component (respectively 67% and 63% of variance). Correlations between eHEALS and age and education were not found. Significant, though weak, correlations were found between the eHEALS and quantity of Internet use (*r* = .24, *P* = .001 and *r* = .24, *P* = .02, respectively). Contrary to expectations, correlations between the eHEALS and successfully completed tasks on a performance test were weak and nonsignificant: *r* = .18 (*P* = .09). The *t* tests showed no significant differences in scores on the eHEALS between participants who scored below and above median scores of the performance test.

**Conclusions:**

The eHEALS was assessed as unidimensional in a principal component analysis and the internal consistency of the scale was high, which makes the reliability adequate. However, findings suggest that the validity of the eHEALS instrument requires further study, since the relationship with Internet use was weak and expected relationships with age, education, and actual performance were not significant. Further research to develop a self-report instrument with high correlations with people’s actual eHealth literacy skills is warranted.

## Introduction

Although a large supply of health information is available to educate and empower people, many lack the capability to use this information for their own benefit [1]. This capability is set out in the concept health literacy, which refers to the ability to read, understand, and communicate about health information to make proper health decisions [2]. In the Netherlands, 11% of the population has low literacy levels, according to the International Adult Literacy Survey [3], and it is assumed that the number of people who have limited levels of health literacy is even higher [4]. In other developed countries this problem is present to the same or worse extent [5,6]. These low levels of (health) literacy are worrisome, since health care is changing, and patients are increasingly expected to be involved in treatment, in health decisions, and in self-management of their disease [7]. As a result, there is an increasing gap between the needed level of health literacy to participate in their own health care, and the actual health literacy level of many patients. Consequently, low levels of health literacy might negatively influence health outcomes, success of treatment, and medical costs [8-10].

### Online Health Information

With the increased diffusion of the Internet among households, the accessibility to relevant health information for the public has increased spectacularly. Controversially, this might also further enlarge the existing differences in health knowledge and access to care [11,12]. After all, collecting information through the Internet is different from collecting information through books and leaflets, and it requires specific skills [13-15]. For example, consumers should be able to use the computer, to navigate their way through the Internet, and to judge the large amount of information in terms of personal relevance, credibility, and accuracy [16]. Because the Internet and its impact keep growing, computer and Internet literacy are becoming an important addition to traditional health literacy skills [17]. Therefore, to get a complete overview of people’s skills to obtain and use health information, we should measure eHealth literacy [11,14,18].

Insight into people’s literacy skills is required to properly deploy guidelines, strategies, and interventions to offer information on different levels and in different formats. This is essential to make health information available and understandable to everyone who needs it [19].

### Measurement of (e)Health Literacy

To measure health literacy levels, the Rapid Estimate of Adult Literacy in Medicine (REALM) [20] and the Test of Functional Health Literacy in Adults (TOFLA) [21] are often used. Both these instruments measure functional health literacy, which implies reading skills and, to some extent, numeracy. Other instruments that tend to measure a broader spectrum of health literacy skills have recently been developed—for example, the Newest Vital Sign [22], the functional, communicative, and critical health literacy scales assessment by Ishikawa et al [23], and the Health Literacy Skills Instrument by McCormack [24]. For the measurement of health-related Internet skills, fewer instruments are available. Recently, Van Deursen and Van Dijk [25,26] proposed an in-depth definition of Internet skills, consisting of operational skills (basic skills to use the Internet), formal skills (navigation and orientation), information skills (finding information), and strategic skills (using the information for personal benefits). This definition derives from the essential combination in eHealth literacy of both technical aspects, related to the use of the Internet, and substantive aspects, related to the content provided by the Internet. The definition contains gradients of difficulty, while the four skills have a sequential and conditional nature [27]. The combination of these four Internet skills illustrates that the application of operational and formal skills alone is not sufficient when using the Internet. On the other hand, using information and strategic skills often depends on the presence of operational and formal skills to obtain information in the first place. All four types of skills can be measured in a series of performance tests in which participants are asked to complete assignments on the Internet (see Multimedia Appendix 1). While this is a valuable method to assess (health-related) Internet skills, it is also quite demanding, costly, and time consuming, which makes it a rather inefficient instrument to use for (clinical) practice and research purposes. Therefore, an easy-to-administer self-assessment instrument that combines the measurement of computer skills with health literacy skills is needed. To our knowledge, the only instrument available that claims to measure the health-related Internet skills of the general Internet user is the eHealth Literacy Scale (eHEALS) by Norman and Skinner [28].

### The eHEALS

The eHEALS is an 8-item scale that tends to measure perceived skills at finding, evaluating, and applying electronic health information to health problems [28]. The instrument proved to be a reliable and easy-to-use self-report tool, and has been used in some studies [29,30]. The scale is based on a model that distinguishes between six types of literacy skills: traditional literacy, health literacy, information literacy, scientific literacy, computer literacy, and media literacy [31]. Accordingly, the eHEALS aims to measure a broad overview of literacy skills, which might make it a potential instrument to assess the effects of eHealth literacy-tailored strategies to deliver online information and applications. However, the eHEALS has until now been available only in English and, to our knowledge, there are no data on its validity. Therefore, the aim of the present study was to examine the reliability and the construct and predictive validity of a Dutch version of the eHEALS.

## Methods

Two populations were studied, one containing patients with rheumatic diseases (study 1) and one containing a stratified sample of the general Dutch population (study 2). Because there are no other instruments that measure eHealth literacy, we measured convergent validity using the associated items age, education, and (health-related) Internet use. Predictive validity was measured by comparison with actual performance on various health-related Internet tasks [32]. Study 1 was originally designed to gain insight into patients’ needs and wishes regarding a Web-based rheumatology patient portal and comprised a survey to measure age, education, general Internet use, health-related Internet use, and the eHEALS [33]. Study 2 was originally meant to gain insight into peoples’ Internet skills and comprised a survey to measure age, education, Internet use, and the eHEALS, plus a series of assignments on an Internet-connected personal computer [32].

### Study 1

#### Population

A random sample of patients with rheumatic diseases was selected from the patient database of the rheumatology clinic of Medisch Spectrum Twente, Enschede, the Netherlands. A total of 496 patients were sent a personal invitation letter and a paper-and-pencil questionnaire by their treating rheumatologists. Patients expected to experience difficulty in completing the survey (e.g. because of significant cognitive impairment or illiteracy) were excluded a priori by their treating rheumatologists. The invitation letter explained the purpose of the study, the use of data, the voluntary nature, and the anonymity of the participant; therefore, returned questionnaires could be presumed to provide consent. A reminder was sent to those who did not respond within 2 weeks. According to local regulations in the Netherlands (Medical Research [Human Subjects] Act) the study did not need approval of the ethical review board; only (nonintervention) studies with a high burden for patients have to be reviewed. For this study, patients who indicated in the questionnaire that they did not have access to the Internet were excluded.

#### Instruments

The questionnaire assessed the following: (1) gender, age, and education level, (2) general and health-related Internet use, and (3) the eHEALS. General Internet use was measured by 2 items: 1 yes/no item measuring access to the Internet, and 1 item on quantity of Internet use with answer options on a 5-point Likert scale ranging from “(almost) never” to “(almost) every day.” Health-related Internet use was measured with 8 items on quantity of use of different kinds of health-related information. Each item could be answered on a 4-point Likert scale ranging from “never” to “regularly” (see Table 1 for a complete overview of topics). The original items of the eHEALS were translated into Dutch with forward and backward translation, according to World Health Organization guidelines [34]. The eHEALS contains 8 items, measured with a 5-point Likert scale with response options ranging from “strongly disagree” to “strongly agree.” Total scores of the eHEALS are summed to range from 8 to 40, with higher scores representing higher self-perceived eHealth literacy. The original version of the eHEALS can be found in Table 2. The whole survey instrument was pretested with 6 participants. Minor revisions were made in formulation and layout according to the received remarks and recommendations.

### Study 2

#### Population

A sample of 88 participants was recruited by randomly dialing telephone numbers in cities and villages in the region of Twente. A stratified sampling method was used to gain equal categories in gender, age, and education. When respondents indicated they were willing to participate, their contact and email address were recorded and a time for the research session was scheduled. All research sessions were scheduled at the University of Twente, which was an unfamiliar environment to all participants. Respondents received a follow-up letter in the mail for confirmation, and the day before the study respondents were reminded of the session by telephone. Respondents were awarded €25 for their participation.

#### Instruments

The sessions lasted approximately 1.5 hours and started off with a short questionnaire that assessed (1) gender, year of birth, and education level, (2) general Internet use, and (3) the eHEALS. General Internet use was measured with 3 items: 1 yes/no item measuring access to the Internet, 1 item measuring amount of Internet use in hours per week, and 1 item on Internet experience in years.

Subsequently, participants had to complete a performance test, which contained nine health-related assignments, based on the four defined Internet skills. Two assignments (consisting of eight tasks) were used to measure operational Internet skills (e.g. open a health website, save a file, or add a website to the Favorites menu), two assignments (consisting four tasks) were used to measure formal Internet skills (e.g. navigate different health-related menu and website designs, and surf between different websites), three assignments were used to measure information Internet skills (find health-related information on the Internet), and two assignments were used to measure strategic Internet skills (e.g. extract information from different sources, and make decisions based on the information found). The assignments were generated by a team of researchers that made a conscious effort to include only tasks that were accessible and relevant to the general user population (e.g. find the Web address of a health clinic, or search for information on vitamins). All assignments were pilot tested with 12 participants to ensure comprehensibility and applicability. Assignments were administered in a sequence of increasing difficulty, as indicated in Multimedia Appendix 1. During the assignment completion, participants themselves decided when they were finished or wanted to give up on an assignment. Completion of the tasks, successful and unsuccessful, was directly noted during the sessions. Tasks were assumed successful if the right answer was given within an ample time period, determined in the pilot tests. To execute the assignments, participants used a keyboard, a mouse, and a 17-inch monitor. The personal computer was connected to the Internet on a high-speed university network and was programmed with the three most popular Internet browsers (Internet Explorer, Mozilla Firefox, and Google Chrome). This allowed the participants to replicate their regular Internet use. No default page was set on the browsers and all the assignments started with a blank page. To ensure that participants were not influenced by a previous user’s actions, the browser was reset after each session by removing temporary files, cookies, and favorites. In addition, downloaded files, history, forms, and passwords were removed and the laptop was rebooted.

### Analysis

Data were analyzed using SPSS version 17.0 for Windows (IBM Corporation, Somers, NY, USA) in both studies. Cronbach alpha served as a measure of internal consistency, reflecting the (weighted) average correlation of items within the scale [35]. In general, a Cronbach alpha of .7 to .8 is regarded as satisfactory for scales to be used as research tools [36]. Principal component analysis was performed to examine the 1-factor structure of the scale. Factor loadings in excess of .71 were considered excellent, .63 very good, and .55 good [37].

Distributional properties of the eHEALS were further inspected to examine the normality of the total scores and to identify floor and ceiling effects. Skewness and kurtosis values between ±1 were assumed to indicate no or slight nonnormality. Floor or ceiling effects were considered to be present if >15% of the participants scored the worst or the best possible score on the eHEALS [38].

Evidence for convergent validity was determined by studying Spearman correlations between total mean scores on the eHEALS and age, education level, quantity of Internet use, and sum scores of health-related Internet use. Based on previous studies on regular health literacy, we hypothesized negative correlations with age and positive correlations with education and (health-related) Internet use [9,11,39]. A coefficient magnitude of at least .4 was taken as evidence of convergent validity [40]. The predictive validity of the instrument was assessed by comparing the total mean scores on the eHEALS with the scores on the actual performance test in study 2, using Spearman correlations. The scores on the eHEALS were first related to the total number of successfully completed tasks. Second, the scores on the eHEALS were related to the amount of completed tasks per skill (operational, formal, information and strategic). A coefficient magnitude of at least .4 was taken as evidence of predictive validity. We used *t* tests on each skill to investigate whether participants who performed below and above the median score of successfully completed assignments significantly differed on the eHEALS. Two-tailed *P* values less than .05 were considered significant.

## Results

### Study 1

#### Participants

Of the 496 invitations sent out, 12 were returned undeliverable. In total, 227 of 484 questionnaires were returned (47%); 189 of these 227 participants had Internet access and completed the eHEALS (83%). Participant characteristics and Internet use are shown in Table 1. Included respondents used the Internet daily or several days a week. Responders and nonresponders did not differ on gender, but nonresponders were on average 5 years younger, with a mean age of 47 years (*P* < .001).

**Table 1:** Participants’ self-reported sociodemographics and (health-related) Internet use

		Study 1 (n = 189) n (%)	Study 2 (n = 88) n (%)
**Gender**			
	Male	119 (63)	45 (51)
	Female	70 (37)	43 (49)
Mean (SD) age (years)		52 (11)	43 (18)
**Education level**			
	Low	38 (20)	25 (28)
	Middle	102 (54)	32 (36)
	High	46 (24)	31 (35)
	Unknown	3 (2)	
**Amount of Internet usage**			
	(almost) Every day	117 (62)	–^a^
	Several days a week	34 (18)	
	About 1 day a week	15 (8)	
	Less than 1 day a week	9 (5)	
	(almost) Never	12 (6)	
	Unknown	2 (1)	
Mean (SD) Amount of Internet use (hours per week)		–^a^	12.2 (13.7)
Mean (SD) Internet experience (years)		–^a^	9.3 (4.3)
**Number of respondents who have ever searched for information on:**			
	Diseases	159 (84)	–^a^
	Healthy lifestyle	121 (64)	
	Medication	95 (50)	
	Treatments	122 (65)	
	Care providers	69 (37)	
	Patient organizations	67 (35)	
	Law regulations related to health conditions	61 (34)	
	Peer-support forums	45 (24)	

^a^ Item was not measured in this study.

#### Distributional Properties

Total scores on the eHEALS were approximately normally distributed with a skewness of -.63. Floor and ceiling effects were acceptable, with no participants scoring the worst possible score (8), and 5 participants scoring the best possible score (40).

#### Reliability and Validity

The internal consistency of the eHEALS was alpha = .93. Unidimensionality of the scale was supported by principal component analysis (eigenvalue = 5.4, 67% of variance explained). The eigenvalue of the first component was 5 times larger than the eigenvalue of the second component (being 1.1). All items loaded high on this component, ranging from .74 to .85 (Table 2). The mean sum score of the scale was 28.2 (SD 5.9).

Table 3 shows the correlations between the scores on the eHEALS and the variables measured in both studies. Correlations with age (*r* = –.11, *P* *=* .13*)* and education (*r* = .09, *P* *=* .24) were not significant. A significant, though weak, positive correlation was found between the eHEALS and quantity of Internet use (*r* = .24, *P* = .001). Concerning health-related Internet use, the use of online information correlated weakly to moderately with the eHEALS with coefficients varying from .26 to .40 (*P* < .001).

**Table 2:** eHealth Literacy Scale (eHEALS) mean items scores, scale reliability, and principal component analysis

Item	Study 1		Study 2		Factor loading		item-total correlation^a^	
	Mean	SD	Mean	SD	Study 1	Study 2	Study 1	Study 2
1: I know what health resources are available on the Internet	3.6	0.83	3.4	0.86	.82	.77	.80	.70
2: I know where to find helpful health resources on the Internet	3.6	0.87	3.3	0.88	.85	.79	.84	.73
3: I know how to find helpful health resources on the Internet	3.7	0.81	3.5	0.94	.85	.86	.85	.72
4: I know how to use the Internet to answer my health questions	3.6	0.85	3.6	0.88	.83	.86	.83	.70
5: I know how to use the health information I find on the Internet to help me	3.5	0.88	3.4	.087	.84	.77	.85	.67
6: I have the skills I need to evaluate the health resources I find on the Internet	3.6	0.89	3.6	0.90	.82	.77	.84	.67
7: I can tell high-quality from low-quality health resources on the Internet	3.4	0.95	3.4	1.00	.80	.75	.82	.76
8: I feel confident in using information from the Internet to make health decisions	3.3	0.99	3.1	1.12	.74	.80	.78	.82
Mean (SD) sum score	28.2	5.9	27.6	5.9				
Eigenvalue first component	5.36		5.06					
Variance accounted for	67%		63%					
Cronbach alpha	.93		.92					

^a^ All item-total correlations were significant at *P* < .001.

**Table 3:** Spearman correlations between scores on the eHealth Literacy Scale (eHEALS) and age, education, (health-related) Internet use, and Internet performance skills

		Study 1		Study 2	
		*r*	*P* value	*r*	*P* value
**Sociodemographics**					
	Age	-.11	.13	-.08	.49
	Education (1 = low, 2 = middle, 3 = high)	.09	.24	.13	.25
	Amount of Internet usage	.24	.001	.24	.02
**Health-related Internet use**					
	Information on diseases	.40	<.001	–^a^	
	Healthy lifestyle	.28	<.001		
	Medication	.29	<.001		
	Treatments	.38	<.001		
	Care providers	.30	<.001		
	Patient organizations	.32	<.001		
	Law regulations related to health conditions	.26	<.001		
	Peer-support forums	.27	<.001		
**Performance tasks**					
	Successfully completed tasks overall	–^a^		.18	.09
	Operational			.12	.27
	Formal			.19	.07
	Information			.05	.62
	Strategic			.11	.30

^a^ Item was not measured in this study.

### Study 2

#### Participants

Characteristics and Internet use of the 88 recruited participants in study 2 are shown in Table 1. Of all participants, 75 (85%) had home Internet access. The average years of Internet experience was 9.3 (SD 4.3) and average amount of Internet use was 12.2 hours a week (SD 13.7).

#### Performance Tests

Table 4 shows that the participants successfully completed an average of 73% (5.8/8) of the operational Internet skills tasks and an average of 73% (2.9/4) of the formal Internet skills tasks. Of the information Internet skills tasks, an average of 50% (1.5/3) was completed successfully and of the strategic Internet skills tasks, 35% (0.7/2). Only 28% (25/88) of the participants were able to successfully complete all operational skills tasks, 39% (34/88) completed all formal skills tasks, 13% (11/88) completed all information skills tasks, and 20% (18/88) completed both the strategic skill tasks. No participants exceeded the maximum amount of time they were given for the assignments. Participants who were not able to complete the assignment decided to give up on the assignment before the official end time had elapsed. More details on the results of the performance tests and the general consequences for health seekers and providers are discussed elsewhere [32].

**Table 4:** Overview of proportion of tasks successfully completed in performance tests

Internet skills (number of tasks)	Average task completion		
	Mean	SD	%
Operational tasks (8)	5.8	2.1	73
Formal tasks (4)	2.9	1.2	73
Information tasks (3)	1.5	0.9	50
Strategic tasks (2)	0.7	0.8	35

#### Distributional Properties

As in study 1, total scores on the eHEALS were approximately normally distributed with a slight skewness of –.80. Floor and ceiling effects were acceptable, with no participants scoring the worst possible score (8), and 4 participants scoring the best possible score (40).

#### Reliability and Validity

The internal consistency of the eHEALS was alpha = .92. All items loaded on 1 single component in this study as well (eigenvalue = 5.1, 63% of variance explained). The eigenvalue of the first component was 5.8 times larger than the eigenvalue of the second component (being .88). All items loaded high on this component, ranging from .75 to .86 (Table 2). The mean sum score of the scale was 27.6 (SD 5.9).

No significant correlations between the eHEALS and either age (*r* = -.08, *P* = .49) or education (*r* = .13, *P* = .25) were found (Table 3). A significant, though weak, correlation was found between the eHEALS and quantity of Internet use (*r* = .24, *P* = .02). The correlations between the eHEALS and actual performance for overall successfully completed tasks and the four skills separately were weak and nonsignificant (Table 3). Comparison on the four performance skills showed that the 50% of participants scoring above the median had a higher mean score on the eHEALS than the 50% of participants scoring below the median (Table 5). However, *t* tests showed that none of these differences were significant (Table 5).

**Table 5:** eHealth Literacy Scale (eHEALS) mean scores of participants scoring below and above median scores on performance tasks

Performance tasks		Mean	SD	*t* test	df	*P* value
**Operational**						
	50% below median	3.38	0.85	–.998	80.33	.32
	50% above median	3.53	0.59			
**Formal**						
	50% below median	3.36	0.77	–1.47	77.38	.15
	50% above median	3.59	0.67			
**Information**						
	50% below median	3.43	0.69	–.26	81.37	.80
	50% above median	3.47	0.80			
**Strategic**						
	50% below median	3.38	0.74	–.79	81.55	.43
	50% above median	3.51	0.74			

## Discussion

The results of the two studies show that the eHEALS is unidimensional and has high internal consistency. Yet results of the validity tests showed that the eHEALS is not a valid measure of eHealth literacy.

With regard to the convergent validity, we hypothesized at least moderate positive correlations (*r* > .4) between scores on the eHEALS and education, and at least moderate negative correlations (*r* > .4) between the eHEALS and age. However, in both studies correlations between the eHEALS and either education or age were not significant. Although it should be noted that (selective) nonresponse might have had an influence, and that younger respondents (<30 years of age) were slightly underrepresented in study 1, we were surprised about the lack of these correlations, as various reviews have shown that these factors are the most predictive for (regular) health literacy [9,39]. In their study, Norman and Skinner [28] found no significant correlation between scores on eHEALS and age either, but in their study only adolescents in the age group of 13–21 years participated. To our knowledge, no other studies have examined the correlation between scores on eHEALS and age and education.

We hypothesized at least moderately positive correlations (*r* > .4) between scores on the eHEALS and quantity of Internet use, since it is reasoned that the amount of time spent on the Internet has a positive influence on eHealth literacy [11]. However, whereas the correlations between the scores on eHEALS and Internet experience were in the expected direction, they appeared to be weak in both of our studies. The correlations between eHEALS and health-related Internet use were weak but slightly higher, with Spearman correlation coefficients ranging from .26 to .40.

Concerning the predictive validity, the lack of significant correlations between the eHEALS and actual performance skills was surprising. Since the assignments used in study 2 were applicable to the general Internet user, one would at least expect some moderate correlations between the eHEALS scale and the performance results. Apparently, perceived skills (as obtained with eHEALS) do not predict actual performance (as measured in study 2). Previous investigations on general computer skills have also shown that people tend to overestimate their computer skills, which results in a gap between self-reported skills and practice when actual skills are measured [41,42]. Furthermore, the comparison of all participants who scored below and above median scores on the performance test did not show any significant differences on the eHEALS either. From this we can conclude that the eHEALS does not have the power to distinguish between people with low health-related Internet skills and people with high health-related Internet skills. These results show that the eHEALS is not a valid instrument for assessing perceived health-related Internet skills.

We suggest a revision of the eHEALS, in a way that all four different skills are measured: (1) operational and (2) formal skills that measure practical use of computers and the Internet, and (3) information finding and (4) strategic skills that measure search strategies and skills to judge the found information. Also, questions might need to be formulated differently in order to prevent misunderstanding or differing interpretations. To this aim, qualitative research might provide more insight into the basis for participants’ answers—for example, having people fill out the eHEALS with techniques such as cognitive interviewing or thinking-aloud methods [43,44]. When measuring al four different skills, we might obtain a more valid indication of eHealth literacy skills. This could also distinguish between what type of skills (groups of) people possess, after which proper implementation of interventions can bring about equal access to online health information for all subgroups.

### Limitations

A limitation of both our studies is the voluntary basis on which participants were recruited. This could have caused a bias, because participants might already have been more interested in using the Internet and searching for information, which could have influenced the results. Concerning study 1, only patients with rheumatic diseases were invited to participate. Therefore, this study might not be representative for other chronic conditions, since patients with rheumatic diseases are on average somewhat older. Concerning study 2, because of the major labor intensity of performance tests and the very high travel costs of bringing participants nationwide to the university lab, it was not possible to test a random sample from the whole Dutch population. Although the study population size of 88 is not enough to generalize to the whole population, the applied quota sample for the categories of gender, age, and education hugely improved representativeness.

### Conclusions

The eHEALS is found to be unidimensional, according to principal component analysis, and to be internally consistent, as assessed with Cronbach alpha, but its validity is questionable. Expected correlations between the eHEALS and peoples’ use of the Internet were weak. Moreover, scores on the eHEALS did not correlate with age, education, and scores on performance tasks, and the eHEALS was not able to distinguish between people with high and low health-related Internet skills. Therefore, more research is needed in order to develop a self-report instrument that validly measures eHealth literacy skills. We suggest incorporation of operational, formal, information, and strategic Internet skills to measure all aspects of eHealth literacy.

## Multimedia Appendix 1

Performance test assignments.
