# Supplementary material for: Does the eHealth Literacy Scale (eHEALS) Measure What it Intends to Measure? Validation of a Dutch Version of the eHEALS in Two Adult Populations
Source: J Med Internet Res. 2011 Nov 9;13(4):e86. doi: 10.2196/jmir.1840 (PMC3222202; doi:10.2196/jmir.1840)
Supplement: Supplementary file 1 [file jmir_v13i4e86_app1.pdf]

Appendix 1: The performance test assignments

**Operational Internet skills**	**Assignment 1** (max time allowed: 12 minutes)
Opening Web sites by entering the URL in the browser's location bar;	1. Go to the Web site of the RIVM (www.rivm.nl).
Using text or images with hyperlinks;	2. Click on the link 'Infectieziekten' in the menu on the left. Click on the subject 'Thema's'. Click on the subject 'Hoofdluis'. Click on the subject 'Voorlichtingsmateriaal downloaden of bestellen'
Opening various common file formats;	3. Open the brochure 'Brochure veelgestelde vragen'
Saving files on the Hard Disk;	Save the brochure in "My Documents".
Navigating forward and backward between pages using the browser buttons;	4. Use the back button to go back to homepage of the RIVM Web site.
Bookmarking Web sites.	5. Add the homepage to the Favourites (or bookmarks)
**Operational Internet skills**	**Assignment 2** (max time allowed: 8 minutes)
Operating Internet-based forms:	6. Go to the Web site of MinVMS (www.minvws.nl).
	Click on the link 'Uitgebreid zoeken.'
Using the different types of fields and buttons;	Complete the fields using the information given.
Submitting a form.	7. Execute the search function and open the third search result.
Saving files from the Internet on the Hard Disk.	8. Save the logo of the MinVWS on the desktop of the computer.
**Formal Internet skills**	**Assignment 3** (max time allowed: 10 minutes)
Not becoming disoriented when navigating within a Web site;	1. Go to the website of ZonMW (www.zonmw.nl).
	Follow the options Onderwerpen / Jeugd / Zorg voor
	Choose the option: 'RIVM/Jeugdgezondheid'.
Not becoming disoriented when navigating between Web sites;	2. In both windows, go to the homepage of the Web site opened:
	Go to the homepage of the RIVM Web site in the new opened window.

Not becoming disoriented when opening and browsing through search results.	Go to the homepage of the RIVM website in the original window.<br><br>3. Perform a search on the ZonMW Web site with the keyword 'infectie'. Open the first search result.<br><br>Open the fourth search result.
**Formal Internet skills**	**Assignment 4** (max time allowed: 10 minutes)
Navigating on the Internet by using hyperlinks (e.g., menu links, textual links, image links) in different menu and Web site layouts.	4. Find the addresses of the following three health organizations. Use the Web sites of the organization:<br><br>ISALA Clinic in Zwolle (www.isala.nl).<br><br>BOSK Organization for the disabled (www.bosk.nl/).<br><br>GGZ Enschede (www.ggznederland.nl)
**Information Internet skills**	**Assignment 5** (max time allowed: 12 minutes)
Locating required information, by:<br><br>Defining search options or queries;<br><br>Selecting information (on a Web site);<br><br>Evaluate information found.	1. Imagine: you would like to know more about the H1N1 influenza. This was originally called the Swine Flu. Answer the following question, using the Web site of Dokterdokter (www.dokterdokter.nl): Why is the name Swine flu not correct?
**Information Internet skills**	**Assignment 6** (max time allowed: 12 minutes)
Locating required information, by:<br><br>Choosing a Web site or a search system to seek information;<br><br>Defining search options or queries;<br><br>Selecting information (in search results);<br><br>Evaluating information sources.	2. Imagine: during a hike you are bitten by a tick. A red spot appears that increases. This is a sign you have been infected with Lyme borreliosis. A friend recommends you to start with an antiviral (remedy against viral infections) immediately, since Lyme's disease can have very unpleasant consequences, especially when treatment starts too late. Answer the following question using a search engine (e.g., Google or the Web site you use at home): Is it a good idea to start an antiviral remedy?
**Information Internet skills**	**Assignment 7** (max time allowed: 12 minutes)
Locating required information, by:<br><br>Choosing a Web site or a search system to seek information;<br><br>Defining search options or queries;<br><br>Selecting information (in search results);<br><br>Evaluating information sources.	3. Imagine: the last few months, your son has been suffering from back problems. His back shows a deviation to the left. It looks like one leg is shorter than the other, although this does not appear to be the case. Answer the following question using a search engine (e.g., Google or the Web site you use at home): What is the name of the condition your son suffers from?

Strategic Internet skills	Assignment 8 (max time allowed: 12 minutes)
Using the Internet, by:<br><br>Developing an orientation towards a particular goal;<br><br>Taking the right action to reach this goal;<br><br>Making the right decision to reach this goal;<br><br>Gaining the benefits resulting from this goal.	1. Imagine: you have a three year old son. Your mother gives you the advise to give him extra vitamins A and D. She believes these are necessary for a healthy growth. Answer the following question using a search engine (e.g., Google or the Web site you use at home): Would you give your son both extra vitamins A and D?
**Strategic Internet skills**	**Assignment 9** (max time allowed: 30 minutes)
Using the Internet, by:<br><br>Developing an orientation towards a particular goal;<br><br>Taking the right action to reach this goal;<br><br>Making the right decision to reach this goal;<br><br>Gaining the benefits resulting from this goal.	2. Imagine: your mother is 82 years old. Lately, she has been suffering from dementia and impaired hearing. You decided to find a homecare organization in Enschede that has a special caring program for these conditions. You also would like the organization to organize daily activities for seniors. Use the Internet to find a homecare organization in Enschede that meets your demands.
